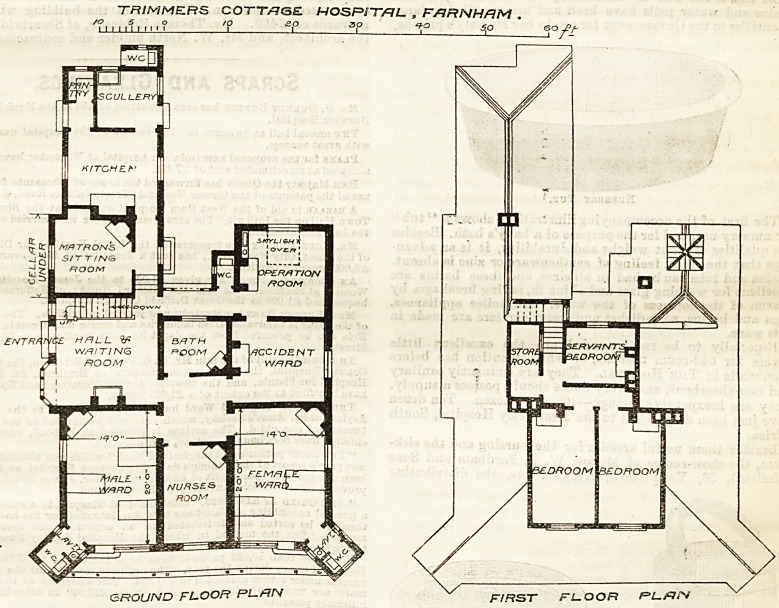# Trimmer's Cottage Hospital, Farnham

**Published:** 1896-02-01

**Authors:** 


### The Institutional Workshop.

### HOSPITAL CONSTRUCTION.

### TRIMMER'S COTTAGE HOSPITAL, FARNHAM.

Two sets of plans appear to have been prepared by Mr. J. A. Eggar, of Farnham, for this building, of which we publish that eventually adopted, presumably on grounds of economy. It is a matter of sincere regret that the first scheme was abandoned, not because the plan as originally proposed was a perfect one, but because it avoided many glaring faults with which the later scheme is crowded. The first plan was conceived on reasonable principles, provided light and air to all parts of the building, and seemed a fairly convenient one. The plans as re-modelled provide an entrance at the west side, directly opening into an outer hall (to be used as a waiting room), from which a staircase leads to the servants' and two other bed-rooms on the upper floor. An inner hall, about 10 ft. 3 in. by 10 ft., opens out of the waiting-room, and has doors opening into two wards, one for either sex, with three beds in each, into a nurses' room between them, and also into a bath-room and into a small accident ward. The matron's room is conveniently placed near the entrance and next the kitchen, which with the usual offices is approached by a passage. A coal place (very small compared with the number of fire-places in the building), ambulance shed, mortuary, and laundry form a separate block of outbuildings. The operating-room—always a difficulty in small hospitals—is connected directly with the accident ward, and, except through this, access can only be obtained to it from the general wards, through the inner and outer halls, and then through a glazed and enclosed corridor, which effectually shuts the open air from the bath-room—a worse arrangement it is difficult to conceive. To bring patients who are to be operated upon in full view of everybody through a number of doors, and round a number of corners, is always objectionable. The inner hall is without direct light, and appears to get no air except what passes to it through the outer hall. The wards are small and narrow; their main light is shadowed by a verandah roof running between the sanitary spur buildings, which do not contain sufficient accommodation for practical work, and their fire-places and doors are so placed as to render the disposition of beds and the warming of the wards a matter of difficulty.

## Figures and Tables

**Figure f1:**